# Effectiveness of Attachment-Based Family Therapy for Suicidal Adolescents and Young Adults: A Systematic Review and Meta-Analysis

**DOI:** 10.32872/cpe.13717

**Published:** 2024-12-20

**Authors:** Poul M. Schulte-Frankenfeld, Josefien J. F. Breedvelt, Marlies E. Brouwer, Nadia van der Spek, Guy Bosmans, Claudi L. Bockting

**Affiliations:** 1 Department of Psychiatry, Amsterdam University Medical Center, University of Amsterdam, Amsterdam, The Netherlands; 2 Department of Pediatric Neurology, Charité – Universitätsmedizin Berlin, Berlin, Germany; 3 Department of Child and Adolescent Psychiatry, Institute for Psychiatry, Psychology & Neuroscience, King’s College London, London, United Kingdom; 4 Centre for Urban Mental Health, University of Amsterdam, Amsterdam, The Netherlands; 5 Department of Clinical Psychology, KU Leuven, Leuven, Belgium; 6 Amsterdam Public Health, Amsterdam University Medical Center, University of Amsterdam, Amsterdam, The Netherlands; Philipps-University of Marburg, Marburg, Germany

**Keywords:** suicide, suicidal ideation, adolescents, young adults, psychotherapy, family therapy

## Abstract

**Background:**

Suicide is a leading cause of death among adolescents and young adults. While only few evidence-based treatments with limited efficacy are available, family processes have recently been posed as a possible alternative target for intervention. Here, we review the evidence for Attachment-Based Family Therapy (ABFT), a guideline-listed treatment targeting intrafamilial ruptures and building protective caregiver-child relationships.

**Method:**

PubMed, PsycINFO, Embase, and Scopus were searched for prospective trials on ABFT in youth published up until November 6^th^, 2023, and including measures of suicidality. Results were independently screened by two researchers following PRISMA guidelines. Risk of bias was assessed using the Cochrane RoB-2 framework. A random effects meta-analysis was conducted on suicidal ideation and depressive symptoms post-intervention scores in randomized-controlled trials (RCTs).

**Results:**

Seven articles reporting on four RCTs (*n* = 287) and three open trials (*n* = 45) were identified. Mean age of participants was *M*_pooled_ = 15.2 years and the majority identified as female (~80%). Overall, ABFT was not significantly more effective in reducing youth suicidal ideation, *g_pooled_* = 0.40, 95% CI [-0.12, 0.93], nor depressive symptoms, *g_pooled_* = 0.33, 95% CI [-0.18, 0.84], compared to investigated controls (Waitlist, (Enhanced) Treatment as Usual, Family-Enhanced Nondirective Supportive Therapy).

**Conclusion:**

Evidence is strongly limited, with few available trials, small sample sizes, high sample heterogeneity, attrition rates, and risk of bias. While not generally superior to other treatments, ABFT might still be a clinically valid option in specific cases and should be further investigated. Clinicians are currently recommended to apply caution when considering ABFT as stand-alone intervention for suicidal youth and to decide on a case-by-case basis.

## Highlights

- Current evidence on the comparative effectiveness of ABFT is strongly limited.
- Meta-analysis showed no improved effect on suicidal ideation nor depressive symptoms compared to other treatments.
- ABFT might still be a clinically valid option in specific cases and should be further investigated.
- Implications and recommendations for researchers and practitioners are discussed in the article.

Suicide is the fourth most prevalent cause of death in adolescents and young adults worldwide (World Health Organization, 2021). In 2019, more than 157.700 youths aged 15 to 29 died prematurely by intentional self-harm, accounting for about 8% of all deaths and the majority of Years of Life Lost in this cohort (Castelpietra et al., 2022; World Health Organization, 2020). Many of these cases are preceded by risk factors and early warning signs such as depressive symptoms, being part of a sexual minority group, expression of suicidal ideas, previous self-harm, or contact with primary health services, and could thus potentially be prevented through targeted treatment (Bachmann, 2018; Chiang et al., 2021; Fergusson et al., 2005; Jackman et al., 2021; Liu et al., 2020; Walby et al., 2018). Several psychological interventions that might help to attenuate the suicidal trajectory are available (e.g., Cognitive Behavioral Therapy (CBT), Dialectical Behavior Therapy (DBT)). However, efficacy of these interventions for adolescent populations was found to be low overall and has stagnated for decades (Busby et al., 2020; Fox et al., 2020; Franklin et al., 2017; Kothgassner et al., 2020; Robinson et al., 2018; Tarrier et al., 2008). Thus, developing more effective interventions to prevent adolescent suicide is warranted (Holmes et al., 2018).

Increasing attention has recently been paid to the role that early childhood interpersonal and relational factors might play in the development and trajectory of mental health problems and suicidal thoughts and behaviors (Chu et al., 2017; Franzoi et al., 2024; Van Orden et al., 2010). Having strong and positive interpersonal connections to family members and friends was found to be protective in this regard, and the absence of such resulting in feelings of loneliness was a risk factor respectively (Gunn et al., 2018; McClelland et al., 2020). At the same time, disturbing aspects of interpersonal relations and familial environments, such as conflicts, constant negative evaluation, childhood trauma, physical abuse, and neglect, can be perceived as major stressors by individuals at risk and contribute to the suicidal trajectory (Carballo et al., 2020; King & Merchant, 2008). Although accumulating evidence suggests that interpersonal relations, specifically in the context of adolescents’ family environments, could be strong targets for preventive interventions, there are only few family-focused interventions available (Frey et al., 2022; Sullivan et al., 2023).

One such family-focused intervention for suicidal youths is Attachment-Based Family Therapy, which targets interpersonal ruptures between youth at risk of suicide and their primary caregivers (G. S. Diamond, 2022; G. S. Diamond et al., 2014). Developed upon the assumption that the quality of familial relations can trigger, exacerbate, and buffer against suicide trajectories, this 16-week treatment protocol addresses interpersonal traumata and dysfunctional interaction patterns in a therapist-guided systematic process. In contrast to currently prevalent treatments mainly targeting patients’ thought patterns and behavior (e.g., Cognitive Behavioral Therapy, Dialectical Behavior Therapy), the five critical treatment tasks in ABFT focus on identifying factors that damaged intrafamilial trust, motivating patients and caregivers to rediscover their innate desire for mutual closeness, and building mature, regulated, and empathic interaction patterns. Adolescents’ autonomy and developmental responsibility are encouraged, and caregivers are supported in developing an empathic, empowering, and unconditionally accepting stance toward their youths. By the end of treatment, attachment security is expected to be improved, building the foundation for future positive development and alleviation of symptoms. A full description of underlying theory and mechanisms can be found in the original manual and a more recent review (G. S. Diamond et al., 2014, 2016).

Attachment-Based Family Therapy is currently listed as a ‘promising’ intervention for youth depression and suicidal ideation at the California Evidence-based Clearing House for Child Welfare, with ratings based on three randomized-controlled trials and four moderation studies published up until 2018 (G. S. Diamond et al., 2002, 2010, 2012; Shpigel et al., 2012; Israel & Diamond, 2013; Feder & Diamond, 2016; Ibrahim et al., 2018; CEBC, 2020). It is further mentioned as a possible intervention for youth suffering from moderate to severe depression in the NICE guidelines (National Institute for Health and Care Excellence, 2019). In recent years, increasing interest in interpersonal treatments for youth suicide bore new evidence, and new results ought to be considered in clinical guidelines and practice. Importantly, more recently published reviews on ABFT remain mostly narrative and do not provide a systematic approach to its evaluation, or do not examine the efficacy of ABFT for youth suicidality specifically (G. S. Diamond et al., 2016, 2021; Sullivan et al., 2023; van Aswegen et al., 2023). One recently published meta-analysis on the effect of family-based treatments in youth finds a significant overall treatment benefit of family-therapy over comparator therapies for suicidal ideation, but not depressive symptoms (Waraan et al., 2023). However, this review pools together different types of family-based therapies (e.g. Family-focused Cognitive Behavioral Therapy, Systems Integrative Family Therapy), thus not allowing to assess the individual efficacy of ABFT. Therefore, this systematic review and meta-analysis aims to summarise and assess the current evidence for the effectiveness of Attachment-Based Family Therapy in treating suicidal adolescents and young adults.

## Method

### Search Strategy and Selection Criteria

This review follows the PRISMA guidelines, was initially performed in August 2021 and updated on November 6^th^, 2023, and preregistered at PROSPERO (CRD42021271731, see Schulte-Frankenfeld et al., 2024S-a). PubMed (Ovid MEDLINE® All 1946 to November 03, 2023), PsycINFO (APA PsycInfo 1806 – October Week 4 2023), and Embase (Embase Classic + Embase, 1946 – 2023 November 03) were searched for trials indexed for the search term “attachment-based family therapy” up until November 06^th^, 2023, using the Ovid interface (version 05.09.00.005). Scopus (November 06, 2023) was searched in titles, abstracts, and keywords. No further filters were applied. Snowballing was performed to identify non-indexed studies by examining published reviews, reference lists of included trials, and contacting expert authors. Search results were deduplicated using ProQuest Refworks.

**Figure 1:**
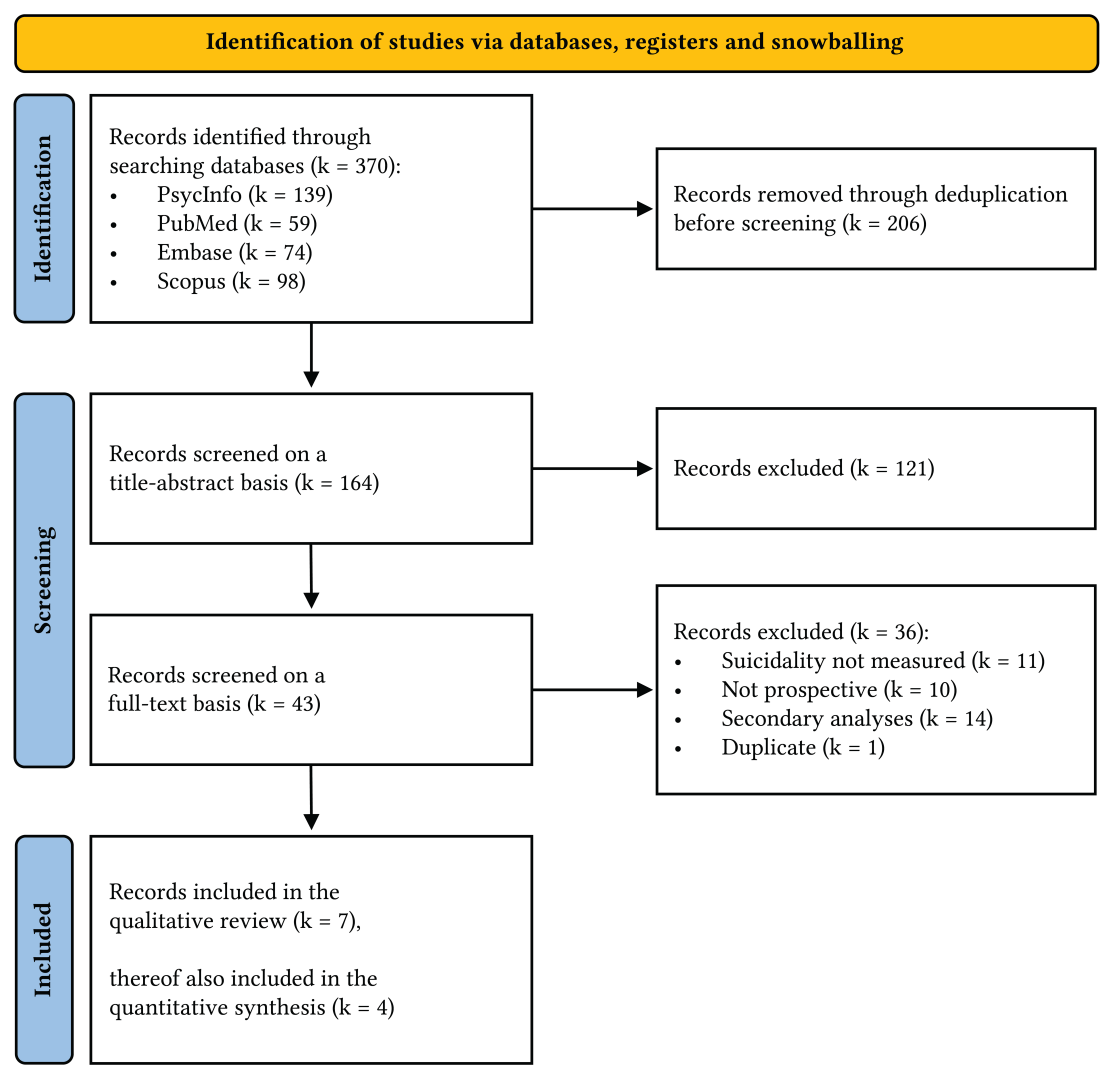
PRISMA Flow Diagram for the Systematic Review

Two reviewers independently screened all records based on titles and abstracts using Rayyan (Ouzzani et al., 2016). Studies were eligible if they fulfilled the following criteria: (1) sample of adolescents and young adults aged ten years and older; (2) ABFT treatment; (3) suicidality measured at post-treatment through diagnostic methods (e.g., clinical interviews, SIQ-JR, BSS, SIDAS); (4) longitudinal or prospective design; (5) written in English, Dutch, or German. Review papers, expert opinions, and case reports were excluded. Potentially eligible records were further assessed on a full-text basis. Risk of bias of eligible records was assessed using the Cochrane RoB-2 framework (Appendix A, Schulte-Frankenfeld et al., 2024S-c). Consensus about deviant decisions was reached by unblinded discussion and consulting a senior author. A list of screened articles (Schulte-Frankenfeld et al., 2024S-b) and a PRISMA Flow Diagram (Figure 1) are available.

### Data Extraction and Statistical Analysis

Primary outcome was the difference in suicidality severity at post-treatment between ABFT and respective control conditions. Further measures of the patient’s idiosyncratic condition (e.g. BDI-II, HAM-D) were collected if available. Demographic information (population type, age, country), study characteristics (*N*, design, conditions), outcome measures, and statistical factors necessary to calculate effect sizes were extracted by one author and independently checked by two others. All authors of included articles were contacted to request Individual Participant Data, and additionally summary statistics when reported outcome data was insufficient to calculate effect sizes. When measurement data from multiple post-baseline timepoints was given, the timepoint most immediate to the end of treatment was used. Recalculating effect sizes based on means and standard deviations was preferred, and data transformations were applied following Cochrane guidelines where necessary (Higgins et al., 2023).

Calculations were performed using RStudio in version 2023.03.0+386 following guidelines by Harrer and colleagues (Harrer et al., 2021; RStudio Team, 2021). *Hedge’s g* was used as effect size measure to correct for small sample bias, expressing the magnitude of difference between group means in units of pooled standard deviation. Although it is commonly interpreted with cut-offs (small (< 0.2), medium (~0.5), and large (> 0.8)) these thresholds are arbitrary and lack solid statistical and empirical foundations. Therefore, they should be interpreted only as rough guidelines within this review (Cuijpers et al., 2014). A random effects model using the Hartung-Knapp method was applied as considerable heterogeneity among studies was expected (van Aert & Jackson, 2019; Zeraatkar et al., 2020). Cochran’s *Q* was used to assess homogeneity (α < .05), and *I*^2^ to quantify how much variability can be attributed to between-study heterogeneity (low: ≤ 25%, medium: 26%–50%, high: > 50%). A prediction interval (95% PI) was calculated to estimate the range in which true effect sizes of future replication studies are expected to fall (IntHout et al., 2016). Publication bias was assessed through visual inspection of colour-enhanced funnel plots. The {tidyverse} package v2.0.0 was used for data processing, and the {esc} package v0.5.1 for calculating effect sizes (Lüdecke, 2019; Wickham et al., 2019). Pooled effect sizes and 95% CIs, measures of heterogeneity, forest plots, and funnel plots were calculated using the {meta} package v6.5-0 (Balduzzi et al., 2019). Risk of bias plots were created using the {robvis} package v0.3.0 (McGuinness, 2019). All data and calculation scripts are publicly available (Schulte-Frankenfeld et al., 2024S-b).

## Results

### Study Selection

PubMed, PsycINFO, Embase, and Scopus were searched for prospective trials indexed for “attachment-based family therapy”, measuring suicidality at post-treatment in a sample of adolescents and young adults aged ten years or older, up until November 06^th^, 2023. Out of 370 records identified, seven articles met the inclusion criteria (*n* = 332), of which four were included in the meta-analysis (*n* = 287) (G. M. Diamond et al., 2012; G. S. Diamond et al., 2002, 2010, 2019; Russon et al., 2022; van der Spek et al., 2024; Waraan et al., 2021). See Figure 1 for the selection process. Individual Participant Data were requested from all authors, though only provided by one team despite repeated outreach attempts. Thus, an Individual Participant Data Meta-Analysis was not performed. One study reported SIQ-JR data deviating between text, figures, and appendix, which only matched after multiplying reported summary scores by the number of items in the underlying scale (Waraan et al., 2021). Another study reported confidence intervals instead of standard deviations, for which a *t*-distribution conversion following the Cochrane Handbook was applied (G. S. Diamond et al., 2010; Higgins et al., 2023).

### Study Characteristics

Characteristics of included studies are depicted in Table 1. Included trials recruited a total of 332 participants (RCTs *n* = 287, Open Trials *n* = 45) with a mean age of 15.2 years (*SD_pooled_* = 1.65, *range:* 12 – 25 years) and the majority identifying as female (~80%). Baseline levels of suicidal ideation, SIQ(-JR), *M_pooled_* = 46.26, *SD_pooled_* = 17.91, and depressive symptoms BDI(-II), *M_pooled_* = 31.59, *SD_pooled_* = 9.00, were high across trials. Heterogeneity between studies regarding populations, treatment protocols, study designs, and implemented control conditions was considerable. Four of the seven studies were randomized-controlled trials using the 12-week (*k* = 2) (G. S. Diamond et al., 2002, 2010) or 16-week (*k* = 2) (G. S. Diamond et al., 2019; Waraan et al., 2021) ABFT protocol, and a waitlist group (*k* = 1) (G. S. Diamond et al., 2002), Treatment as Usual (TAU) (Waraan et al., 2021), directly referred “enhanced” usual care (Enhanced Usual Care; EUC) (G. S. Diamond et al., 2010), or a nondirective supportive therapy augmented with psycho-educative sessions for primary caregivers (Family-Enhanced Nondirective Supportive Therapy; FE-NST) (G. S. Diamond et al., 2019) as control condition. TAU and EUC trials did not follow-up participants for the type of treatment received (G. S. Diamond et al., 2010; Waraan et al., 2021). The open non-controlled trials consisted of one study using the 16-week ABFT protocol, and two applying LGBTQ+ sensitive variants of ABFT to sexual minority youths (G. M. Diamond et al., 2012; Russon et al., 2022; van der Spek et al., 2024). Clinically significant scores of suicidal ideation were a recruitment criterion in five studies (RCTs *k* = 2; Open Trials *k* = 3), while two RCTs primarily recruited adolescents with depressive symptoms (G. S. Diamond et al., 2002; Waraan et al., 2021). Overall, trials were small, with samples ranging from 10 to 129 participants. A priori calculated recruitment targets were only met by one study (G. S. Diamond et al., 2019). One study presented a post-intervention attrition rate of ~80%, raising concerns about its validity (Waraan et al., 2021). After conducting a sensitivity analysis, we kept it for completeness. Risk of bias was high in most studies, with non-blinded assessment and selective data reporting as major issues (Appendix A and B, Schulte-Frankenfeld et al., 2024S-c).

### Effect Sizes and Meta-Analyses

Suicide attempts and suicidal ideation, as assessed through the Suicidal Ideation Questionnaire (SIQ), were the only shared measure of suicidality between studies. Since the number of recorded attempts was too low for statistical analysis (see Table 1), SIQ scores were used to compare effect sizes.

**Table 1:** Characteristics of Studies Included in the Systematic Review

Study	Population	Inclusion criteria	*N*	Age, *M (SD, range)*	Gender, % Female	Location	Design	Conditions	Measure of effect	Secondary outcomes	Attrition rate	Adherence, *M* (*SD*, *range*)	Baseline Severity, *M* (*SD*)	Effect Size,*g*, [95% CI]	Clinical Recovery	Suicidal Behavior	Conclusion	Risk of Bias
Diamond et al., 2002 ^a^	Depressed youth, referred by schools and caregivers	MDD primary diagnosis according to DSM-III-R	32	14.9 (1.5, *range*: 13 - 17)	78%	USA	Pilot RCT	*ABFT:* 12 weeks ABFT *Control:* 6 weeks waitlist	SIQ	BDI, HAM-D, BHS, STAIC	3%	*NA*	*SIQ:* 32.10 (20.37) *BDI:* 25.9 (7.45)	*time*: 0.66 [-0.05, 1.38] *group*: 0.37 [-0.34, 1.08]	*NA*	*NA*	No effect over time, not superior to waitlist	High
Diamond et al., 2010 ^a^	Suicidal youth, referred by primary care and emergency rooms	SIQ-JR > 31, BDI-II > 20	66	15.1 (1.5, *range*: 12 - 17)	83%	USA	RCT	ABFT: 12 weeks ABFT *Control:* 12 weeks EUC	SIQ-JR	SSI, BDI-II	9%	*ABFT:* 9.71 (5.26) *Control:* 2.87 (3.3) Significant group difference (*Z* = -4.74, *p* < .001)*	*SIQ-JR:* 51.07 (13.45) *BDI-II:* 33.00 (9.03)	*time*: 3.91 [3.08, 4.74]* group: 0.83 [-0.30, -1.32]*	Remission (≤ 13): 87.1% *OR* = 6.30 [1.76 – 22.61]*	Suicide attempts (up to 6 months after baseline): *ABFT:* 11% (*n* = 4) *Control:* 23% (*n* = 7)	Effect over time, superior to EUC	High
**G. M. Diamond et al., 2012**	Suicidal LGB youth	LGB identification, SIQ-JR ≥ 31	10	15.1 (1.37, *range*: 14 - 18)	80%	USA	Open Trial	*ABFT:* 12 weeks ABFT-LGB	SIQ-JR	BDI-II	20%	*ABFT: NA* (*range*: 8 – 16)	*SIQ-JR:* 51.00 (13.00) *BDI-II:* 28.10 (13.63)	*time*: 3.86 [2.24, 5.47]*	Remission (≤ 13): 87.5%	*NA*	Effect over time	*NA*
Diamond et al., 2019 ^a^	Suicidal youth, referred by primary and emergency care, inpatient clinics, schools, and self-referral	SIQ-JR ≥ 31, BDI-II > 20	129	14.87 (1.68, *range*: 12 - 18)	81.9%	USA	RCT	*ABFT:* 16 weeks ABFT *Control:* 16 weeks FE-NST	SIQ-JR	BDI-II	18%	*ABFT:* 14.34 (7.58) *Control:* 12.67 (5.74) No significant group difference, *t*_127_ = -1.43, *p* = .16	*SIQ-JR:* 49.83 (15.08) *BDI-II:* 30.59 (7.94)	*time*: 1.91 [1.47, 2.34]* group: 0.17 [-0.21, 0.55]	Remission (< 12): 32.7% vs. 24.4%	Suicide attempts (during treatment): *ABFT:* 3% (*n* = 2) *Control:* 8% (*n* = 4)	Effect over time, not superior to FE-NST	Some concern
Waraan et al., 2021 ^a^	Depressed youth, referred to mental health clinics	Current MDE according to clinical interview, GRID-HAMD > 15	60	14.9 (1.35, *range*: 13 - 18)	87%	Norway	RCT	*ABFT:* 16 weeks ABFT *Control:* 16 weeks TAU	SIQ-JR	GRID-HAMD, BDI-II	80%	*NA*	*SIQ-JR:* 43.09 (22.96) *BDI-II:* 35.22 (8.66)	*time*: 0.64 [-0.32, 1.61] *group*: 0.10 [-0.66, 0.86]	*NA*	*NA*	No effect over time, not superior to TAU	High
**Russon et al., 2022**	Suicidal LGBTQ+ youth	LGBTQ+ identification, SIQ-JR ≥ 31, BDI-II ≥ 21	10	18.2 (*NA*, *range*: 15 - 25)	NA	USA	Open Trial	*ABFT:* 16 weeks ABFT-LGBTQ+	SIQ-JR	BDI-II	0%	*ABFT:* 15.3 (*range*: 11 – 23)	*SIQ-JR:* 52.70 (19.36) BDI-II: 35.40 (11.92)	*time*: 1.45 [0.40, 2.50]*	Remission (≤ 13): 11%	Suicide attempts (during treatment): *ABFT:* 11% (*n* = 1)	Effect over time	*NA*
**van der Spek et al., 2024**	Depressed and/or suicidal youth referred to an outpatient clinic	Clinical assessment, depressive and suicidal symptoms	25	17.1 (*NA*, *range*: 12 - 23)	74.2%	Netherlands	Open Trial	*ABFT:* 16 weeks ABFT	SIQ-JR	CDI-2	32%	*ABFT:* 89% completed 10 or more sessions	SIQ-JR: 36.31 (11.93) CDI-2: 32.07 (11.48)	*time*: 0.72 [0.09, 1.35]*	*NA*	Suicide attempts (during treatment): *ABFT:* 14% (*n* = 4)	Effect over time	*NA*

*Note.* Effect sizes for primary outcomes calculated as within-group effect over time for the ABFT group (*time*) and between groups at post-treatment (*group*). Sample size (*N*) represents the total number of participants included in the respective trial, the number of available datapoints per measure used to calculate effect sizes can deviate. *ABFT* indicates values for the ABFT intervention group, *Control* for the control group respectively. Attrition rate was calculated as the number of datapoints at post-treatment compared to baseline for the SIQ(-JR) scale as the primary outcome. Adherence was assessed as the amount of completed treatment sessions. Baseline severity represents outcome statistics for the total sample estimated from group-wise baseline scores. ABFT = Attachment-Based Family Therapy; ABFT-LGB = LGB sensitive variant of ABFT; ABFT-LGTBQ+ = LGBTQ+ sensitive variant of ABFT; BDI-III = Beck Depression Inventory-II; CDI-2 = Children’s Depression Inventory 2^nd^ Edition; EUC = Enhanced Usual Care; FE-NST = Family-Enhanced Nondirective Supportive Treatment; GRID-HAMD = GRID Hamilton Rating Scale for Depression; LGB = lesbian, gay and bisexual; MDD = Major Depressive Disorder; MDE = Major Depressive Episode; NA = not available; SIQ-JR = Suicidal Ideation Questionnaire-Junior; SSI = Scale for Suicidal Ideation; TAU = Treatment as Usual. ^a^Studies included in the meta-analysis. *confidence interval excluding zero indicating significance or *p* < .05.

The SIQ scale is a commonly applied self-administered questionnaire appropriate for adolescents around Grade 10 – 12, consisting of 30 items measuring mental distress and suicidal intend. Participants receive a list of “thoughts that people sometimes have” and are instructed to “indicate which of these thoughts [they] have had in the past month” on a scale from 0 “I never had this thought” to 6 “almost every day” (Reynolds, 1987). The SIQ-JR scale is an adapted version of the SIQ, consisting of a 15-item subset appropriate for adolescents around Grade 7 – 9 (Reynolds & Mazza, 1999). Scores consist of summed item responses, thus ranging from 0 to 180 for the SIQ and 0 to 90 for the SIQ-JR respectively, with higher scores indicating higher levels of mental distress and suicidal intend.

Out of seven studies, four trials presented moderate to large within-group changes in SIQ(-JR) scores over time, *g* = 0.72 to 3.91 (G. M. Diamond et al., 2012; G. S. Diamond et al., 2002, 2019; van der Spek et al., 2024). When compared with controls at post-treatment, a significant difference between treatment and comparator groups was present in one out of four RCTs, *g* = 0.83, 95% CI [0.30, 1.36], whereby the pooled effect size was large and reported to remain stable after 24 weeks (G. S. Diamond et al., 2010). Trials utilizing other comparators did not reveal significant group differences in SIQ(-JR) scores at post-treatment, and follow-up data was unavailable (G. S. Diamond et al., 2002, 2019; Waraan et al., 2021).

**Figure 2:**
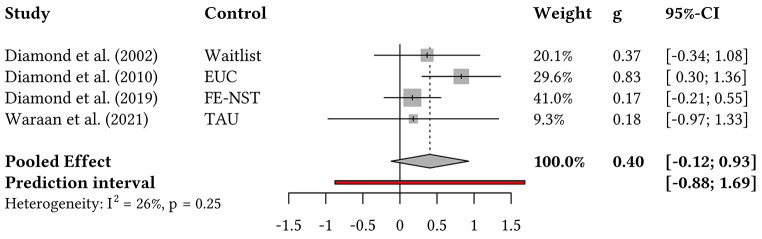
Forest Plot on the Effect of ABFT Versus Control Treatments for Reducing SIQ(-JR) Scores *Note.* Effect sizes for SIQ(-JR) scores calculated between groups at post-treatment as Hartung-Knapp corrected Hedges’ *g* values. EUC, Enhanced Usual Care; FE-NST, Family-Enhanced Nondirective Supportive Therapy; TAU, Treatment as Usual.

A meta-analysis was performed on SIQ(-JR) scores. Four trials (*n* = 287) were included in this synthesis, the results of which are depicted in Figure 2. The pooled effect size was *g_pooled_* = 0.40 with a confidence interval including zero, 95% CI [-0.12, 0.93], indicating that SIQ(-JR) scores of participants in the ABFT conditions did not significantly differ from those in the associated control conditions at post-intervention. The prediction interval spanning zero, 95% PI [-0.88, 1.69], suggests that ABFT will likely not benefit some patients in future trials with similar conditions based on current evidence. Variation between effect sizes attributed to study heterogeneity was moderate, with *I*^2^ = 26% and Cochran’s Q being non-significant, *p* = 0.25. Visual inspection of the colour-enhanced funnel plot (Appendix C, Schulte-Frankenfeld et al., 2024S-c) did not indicate asymmetry. A sensitivity analysis restricted to active-control trials (EUC, FE-NST, TAU) yielded similar results, *g_pooled_* = 0.42, 95% CI [-0.55, 1.39], *I*^2^ = 51%, and Cochran’s Q being non-significant, *p* = 0.13. Subgroup analyses were not performed due to the low number of included studies (Cuijpers et al., 2021).

Since all studies also measured the intervention's effect on depressive symptoms, performing a secondary meta-analysis on BDI(-II) scores was possible. The Beck’s Depression Inventory (BDI) and its revised version, the BDI-II scale, are widely used 21-item self-report rating inventories measuring characteristic attitudes and symptoms of depression (Beck, 1961; Beck et al., 1996). Ratings per item range from 0 (not present) to 3 (strongly present) and scores are calculated by summing all item ratings, with higher scores indicating higher levels of depressive attitudes and symptoms. In this meta-analysis of post-intervention BDI(-II) scores across trials, the pooled effect size was *g_pooled_* = 0.33 with a confidence interval including zero, 95% CI [-0.18, 0.83], indicating that BDI(-II) scores of participants in the ABFT condition did not significantly differ from those in the associated control conditions. The prediction interval includes zero, 95% PI [-0.83, 1.49]. Heterogeneity was moderate, with *I*^2^ = 24% and Cochran’s Q non-significant, *p* = 0.27. A sensitivity analysis restricted to active-control trials (EUC, FE-NST, TAU) yielded similar results, *g_pooled_* = 0.23, 95% CI [-0.43, 0.88], *I*^2^ = 2.8%, Cochran’s Q *p* = 0.36.

## Discussion

This study is the first to systematically review the efficacy of Attachment-Based Family Therapy (ABFT), a psychotherapeutic treatment for pediatric patients focussing on intrafamilial attachment dynamics, for treating youth who are suicidal. Out of 370 records, four randomized-controlled trials (*n* = 287) and three open trials (*n* = 45) measuring suicidality were identified. In the meta-analysis covering four RCTs, Attachment-Based Family Therapy was not superior in reducing suicidal ideation or depressive symptoms compared to investigated controls (waitlist, FE-NST, EUC, TAU), and these results remained stable when restricting the analysis to active control interventions only. Nonetheless, most studies (5 out of 7) reported significant reductions in suicidal ideation and depression within the ABFT treatment group over time.

Overall, experimental evidence is limited, with few available trials, small sample sizes, limited follow-up data, high sample heterogeneity, attrition rates, and risk of bias. Currently, only four controlled trials including 287 participants exist, which might be insufficient to robustly establish or rule out superiority in a comparison with anticipated small effect sizes. Follow-up data was only available for one out of four RCTs, limiting the interpretability of clinical effects and their sustainability over time (G. S. Diamond et al., 2010). A priori defined recruitment targets were only met in one trial, indicating that most studies were underpowered (G. S. Diamond et al., 2019). Loss-to-follow-up was heterogeneous, with one study exhibiting a pre-post loss rate of ~80% for the primary outcome (Waraan et al., 2021). Attrition rates in the other randomized-controlled trials were moderate (3%–18%) and in line with estimates from other psychotherapy trials for depression in children and youth (Cooper & Conklin, 2015; Wright et al., 2021). SIQ(-JR) and BDI(-II) scales were used in most trials to assess suicidality and depressive symptoms, introducing some risk of instrument bias. Additionally, the SIQ(-JR) scale has recently been criticized for its insufficient psychometric properties (Courtney et al., 2024). Emerging evidence further suggests that severity of suicidal ideation strongly fluctuates short-term, for which multiple assessments might be necessary to measure state suicidality reliably (Czyz et al., 2019; Kleiman et al., 2017). There was also considerable design heterogeneity between trials. Two studies in the meta-analysis used the 12-week ABFT protocol, while others applied the treatment for 16 weeks. Comparator treatments differed strongly, with trials using waitlist control, (enhanced) Treatment as Usual (EUC, TAU), or Family-enhanced Nondirective Supportive Therapy (FE-NST), which interestingly has been found to be effective for adult depression (Cuijpers et al., 2012). Actual type of treatment delivered was not assessed and adequately described in EUC and TAU trials, which might contribute to unexplained heterogeneity (Burns, 2009; Witt et al., 2018). Further so, three out of four included RCTs were conducted by researchers associated with the treatment development group. Primary allegiance of care providing professionals was only reported in one RCT, potentially introducing allegiance bias. Unfortunately, extending the meta-analysis through subgroup analyses for heterogeneity factors was not feasible due to the limited number of included trials and lack of Individual Participant Data. As a result, it was not possible to assess the respective impact of these sources of bias on the potential underestimation or overestimation of the comparative effect.

Narrative reviews of previous trials suggested that Attachment-Based Family Therapy might be more effective in reducing suicidal ideation and depressive symptoms in youth than the current standard of care, which was not confirmed by this meta-analysis (G. S. Diamond et al., 2016, 2021; Ewing et al., 2015). Although restrained in validity due to the described limitations, these findings are in line with systematic reviews on the effect of other family-based interventions on depressive symptoms and a large meta-analysis on the effect of all-type treatments (e.g., medication, psychotherapy, combined) on several measures of suicidality (e.g., ideation, (non-)suicidal self-injury, death) in adolescents, which neither found significant treatment effects, regardless of outcome measure and intervention type (Harris et al., 2022; van Aswegen et al., 2023; Waraan et al., 2023). Similar patterns can be observed with treatments for youth depression, which generally tend to yield lower effects than interventions for adults, and to which a substantial amount of patients do not respond within time (Cuijpers et al., 2020, 2023). For preventative approaches, data availability is currently strongly limited, and individual reports suggest that universal approaches might even lead to adverse effects on at-risk youth (Breedvelt et al., 2018; Montero-Marin et al., 2022). In contrast to these results, it should be noted that one other meta-analysis did identify a small positive effect of various family-based interventions on youth suicidal ideation, which upon closer inspection appears to be due to a discrepancy in data extraction for one specific trial (Waraan et al., 2023). As retrieving underlying data from this meta-analysis was not possible, we verified our extracted data with the author team of the trial in question to ensure correctness. Overall, effectiveness of current treatment options for youth suicidality is strongly limited and a major concern in pediatric healthcare.

Considering the high burden of disease implicated by continued suicidal ideation, attempts, and completed suicide in youth, developing more effective treatments is imperative. Previous discussions of this challenge suggested that integrating insights on the dynamics of youth, characterized by strong psychological, biological, and social volitions, might improve efficacy (Harris et al., 2022; Robinson et al., 2018). While Attachment-Based Family Therapy was, in contrast to more commonly applied psychological interventions (e.g., Cognitive Behavioral Therapy, Dialectical Behavior Therapy), developed explicitly for a pediatric population, it being non-superior to compared alternative treatments raises the question if other factors, such as patient baseline characteristics or therapeutic modalities, might influence treatment efficacy. Recent investigations on moderating factors in previous ABFT trials suggest, that patients with higher baseline levels of parent-teen conflict and underserved family backgrounds particularly benefit from treatment, and that more change in family cohesion during the intervention period was related to better treatment outcomes (Ibrahim et al., 2022; Zisk et al., 2019). Targeting Attachment-Based Family Therapy at high-yield subgroups, e.g., LGBTQI+ youth with non-accepting parents, could thus be beneficial (G. M. Diamond et al., 2022; Russon et al., 2023). Another area of improvement could be to intensify treatment components contributing to family cohesion, and disrupt negative feedback dynamics early on by e.g. implementing supervised exposure exercises to reduce patients’ fear of the caregiver’s emotional rejection (Bosmans et al., 2022). Other reviews pointed out that participants with higher baseline levels of depression, non-suicidal self-injury, perceived burdensomeness, and anxiety profited less from the treatment (Abbott et al., 2019; Herres et al., 2021). This might indicate that other interventions could have suited these patients better, or that sequential or multimodal approaches addressing their comorbidities and family dynamics simultaneously might have had better effects, e.g., combining Attachment-Based Family Therapy and Cognitive Behavioral Therapy for patients with anxiety (Herres et al., 2023). Considering given evidence for moderating factors, it is thus conceivable that ABFT might potentially lead to better treatment outcomes compared to the current standard of care when applied to patients whose condition is strongly linked to dynamics of intrafamilial relations, and whose comorbid disorders are concurrently addressed – that is, when the right patient receives the right treatment at the right time.

Given the limitations of current evidence on the efficacy of Attachment-Based Family Therapy for treating youth who are suicidal, future research should focus on delivering high-quality evidence with more adequately powered samples, longer follow-up times, and more consistent and rigorous measurements. At the time of review, two adequately powered randomized-controlled clinical trials evaluating its performance and cost-effectiveness were registered at ClinicalTrials.gov (Bockting et al., 2023; Pachankis et al., 2023). Additionally, with the weight of evidence suggesting that ABFT yields varying degrees of benefit for different patient groups – on average not exceeding the effectiveness of alternative interventions such as TAU or FE-NST – a better understanding of underlying active ingredients and treatment-induced mechanisms of change might be helpful to dismantle contexts and conditions under which specific treatment components lead to better treatment outcomes (Cuijpers et al., 2019; Hofmann & Hayes, 2019). Previously, limited evidence for the role of therapeutic alliance as a common factor, and guided emotional processing, improved parenting, and reattachment as treatment-specific factors was found (G. S. Diamond et al., 2021). Future research could further focus on disentangling these specific factors from common factors also activated in alternative treatments such as TAU or FE-NST, understanding the underlying processes of change involved, identifying therapeutic components that activate these changes in a cost-efficient manner, and providing clinicians with informed guidelines on implementing these components within a process-based therapy framework (Hofmann & Hayes, 2019; Rief et al., 2024).

Finally, a key challenge is to establish predictive factors for when individual treatment components of Attachment-Based Family Therapy might be feasible for a patient. While tailoring therapy plans and manualized treatments to the needs of individual patients and their presenting condition is common practice among clinicians, the application of prediction and personalization models in research remains scarce (Meehan et al., 2022). Meanwhile, research indicates that such personalized medicine approaches could result in substantial benefits for patients, and it might therefore be valuable to investigate further ‘what works for whom’ when it comes to dynamicity-embracing treatments for suicidal youth (Deisenhofer et al., 2024; DeRubeis et al., 2014; Robinson et al., 2018). Ultimately, prioritizing investment into larger cohort studies and analyses of Individual Patient Data to develop and improve targeted preventative approaches in adolescence is crucial, as this special developmental stage provides a unique window of opportunity to stimulate long-lasting positive life trajectories (Breedvelt et al., 2018, 2024; Dahl et al., 2018).

## Supplementary Materials

The Supplementary Materials contain the following items:

- The preregistration for the study (Schulte-Frankenfeld et al., 2024S-a).
- All collected summary statistics per study, analysis scripts, and a list of all articles screened (Schulte-Frankenfeld et al., 2024S-b)
- Online appendices: Additional information on the meta-analysis, namely detailed risk-of-bias scores per study and category, weighted overall risk-of-bias scores, and a color-enhanced funnel plot on the distribution of effect sizes of included studies [Supplements 1] (Schulte-Frankenfeld et al., 2024S-c).

Schulte-FrankenfeldP. M.
BreedveltJ. J. F.
BrouwerM. E.
van der SpekN.
BosmansG.
BocktingC. L.
 (2024S-a). The effectiveness of attachment-based family therapy for suicidal adolescents and young adults: A systematic review and meta-analysis
[Preregistration]. PsychOpen. https://www.crd.york.ac.uk/prospero/display_record.php?ID=CRD42021271731
10.32872/cpe.13717PMC1196057340177611

Schulte-FrankenfeldP. M.
BreedveltJ. J. F.
BrouwerM. E.
van der SpekN.
BosmansG.
BocktingC. L.
 (2024S-b). Attachment-based family therapy for suicidal adolescents and young adults: A systematic review and meta-analysis
[Summary statistics, analysis scripts, list of screened articles]. PsychOpen. 10.17605/OSF.IO/2SWE8
PMC1196057340177611

Schulte-FrankenfeldP. M.
BreedveltJ. J. F.
BrouwerM. E.
van der SpekN.
BosmansG.
BocktingC. L.
 (2024S-c). Supplementary materials to "Effectiveness of attachment-based family therapy for suicidal adolescents and young adults: A systematic review and meta-analysis"
[Online appendices]. PsychOpen. 10.23668/psycharchives.15566
PMC1196057340177611

## Data Availability

All data and calculation scripts for this study are publicly available (see Schulte-Frankenfeld et al., 2024S-b).

## Preregistration

This systematic review was preregistered at the International Prospective Register of Systematic Reviews, PROSPERO: CRD42021271731.

## Reporting Guidelines

This systematic-review and meta-analysis was conducted in accordance with the PRISMA guidelines. The Cochrane Rob-2 framework was applied to assess the risk of bias in randomized-controlled trials.

## Social Media Accounts

@poschfeld, @JosefienUMH, @clbockting, @mebrouwer

## Abbreviations

ABFT – Attachment-Based Family Therapy

CBT – Cognitive Behavioral Therapy

DBT – Dialectical Behavior Therapy

EUC – Enhanced Usual Care

FE-NST – Family-Enhanced Nondirective Supportive Therapy

TAU – Treatment as Usual
